# The diagnostic pathway of Alzheimer's disease in real-world clinical practice in Spain: results from the Adelphi Dementia Disease Specific Programme™

**DOI:** 10.3389/fneur.2025.1702805

**Published:** 2026-01-20

**Authors:** Pascual Sánchez-Juan, Pablo Baz, Enrique Arrieta, Diego Novick, Silvia Díaz-Cerezo, Sarah Cotton, Chloe Walker, Ángel Trueba Saiz, Mercedes Núñez

**Affiliations:** 1 Reina Sofía Alzheimer Center, CIEN Foundation, Instituto de Salud Carlos III, Madrid, Spain; 2 Centro de Investigación Biomédica en Red de Enfermedades Neurodegenerativas (CIBERNED), Instituto de Salud Carlos III, Madrid, Spain; 3 Centro de Salud Periurbana Norte, Salamanca, Spain; 4 Centro de Salud Segovia Rural, Segovia, Spain; 5 Eli Lilly & Company, Arlington, United Kingdom; 6 Eli Lilly & Company, Alcobendas, Spain; 7 Adelphi Real World, Bollington, United Kingdom

**Keywords:** Alzheimer's disease, biomarkers, early diagnosis, mild cognitive impairment, neurocognitive disorders

## Abstract

**Introduction:**

There are challenges associated with the timely diagnosis of people with mild cognitive impairment (MCI) and Alzheimer's disease (AD), especially with the future introduction of amyloid-targeting therapies. This study evaluates the current diagnostic journey for MCI and dementia due to AD in Spain.

**Methods:**

This study used data from the Adelphi Real World Dementia Disease Specific Programme (DSP^TM^), a cross-sectional survey with retrospective data collection. The survey involved primary care physicians (PCPs) and hospital specialists with experience in managing and treating AD, along with their consulting patients, between January and July 2023 in Spain. Analyses were descriptive.

**Results:**

Physicians (*N* = 94; 44.7% PCPs, 38.3% neurologists) reported data for 723 patients. Most patients (78.9%) first consulted a PCP. The median (inter-quartile range) time since symptom onset to first consultation was 21.6 (6.1–48.2) weeks and, in those patients who were not diagnosed immediately, the time from first consultation to diagnosis was 13.0 (6.0–21.9) weeks if diagnosed by a PCP, or 28.9 (17.1–52.1) weeks if diagnosed by a specialist. The diagnosing physician was a specialist or a PCP for 83.2% and 16.8% of patients, respectively. In addition, the most used advanced diagnostic techniques were computed tomography scans and magnetic resonance imaging (57.2% and 43.3% of patients, respectively). CSF determination of AD biomarkers was conducted in 12.4% of patients and AD-specific blood biomarkers in 2.9%. Treatment was mostly initiated by neurologists.

**Conclusion:**

The diagnostic process for people with MCI and AD could be accelerated by increased awareness of the disease, shorter referral times, and better access to specialized diagnostic services. This study shows limited use of AD-specific biomarker testing in Spain.

## 1 Introduction

Alzheimer's disease (AD) is a chronic and progressive neurodegenerative condition that ranges from a preclinical stage to mild cognitive impairment (MCI) and, finally, a phase of dementia (1, 2). Epidemiological studies have shown that most people with early-stage AD (MCI and dementia due to mild AD) remain undiagnosed, and misdiagnoses are common (3, 4). The reason is, in part, due to the diagnosis of AD being based exclusively on a clinical evaluation (5). The development of blood-based biomarkers and *in vivo* biomarkers, such as those based on cerebrospinal fluid (CSF) and positron emission tomography (PET), offer the potential for a more accurate and timely diagnosis of AD (6). Better management of risk factors can help take preventive lifestyle measures and care planning, and anticipate related pathologies (1, 7). In addition, the identification of suitable patients who may benefit from amyloid-targeting therapies as soon as possible after the first symptoms of MCI and dementia due to mild AD is essential (7–10).

In Spain, more than 900,000 people (2.2% of the total population in 2022) are estimated to be affected by AD, with approximately 40,000 new cases diagnosed annually (11, 12). However, accurate data on the epidemiology of the various stages of AD remain unavailable. A recent study showed that the prevalence of treated people with AD was estimated at 760.5 per 100,000 inhabitants (13). In addition to the large numbers of people with AD, the economic impact of dementia in the healthcare system and in society at large is huge and it is estimated to greatly increase with the progressive aging of the population (12). Since primary care is the entry point to the health system in Spain for people with MCI and AD, it is relevant to understand the factors that determine the identification, referral, and management of these patients (14). It is also unclear what specific symptoms drive the search for medical attention in people with AD or their caregivers at its early stages (15, 16), and the impact of diagnosis (17). AD diagnosis can be a multi-step and lengthy process involving several distinct healthcare providers and settings (18–20). Despite the availability of biomarkers and their associated technologies in Spain, an overloaded healthcare system has been identified as a barrier to speedier diagnosis of suspected patients (15). Understanding the current diagnostic process of people with MCI and AD in the Spanish healthcare system is of vital importance to identify areas for improvement.

A recent study evaluated the diagnostic journey and the challenges associated with the timely diagnosis of people with MCI and AD in real-world clinical practice in multiple countries, including Spain (21). This study was based on survey data of physicians relating to patients under their care with MCI and AD. Here, we present a more detailed analysis of the characteristics of the Spanish cohort, focusing on the clinical characteristics of people with MCI and AD and their clinical presentation, from primary care to hospital care, including the main diagnostic tests used and their associated waiting times. The study also analyzed the key factors that influence the search for medical help by people with MCI and AD in Spain, including symptoms, the healthcare professional who is initially consulted, and the causes of the delay in seeking medical help.

## 2 Materials and methods

Data were drawn from the Adelphi Real World Dementia Disease Specific Programme (DSP^TM^), a cross-sectional survey with retrospective data collection of primary care physicians and specialists with experience in managing and treating AD, and their consulting patients, between January and July 2023 in Spain. The DSP methodology has been previously published, validated (22–24), and found to be consistent over time (25). Data were collected anonymously and aggregated, ensuring that neither patients nor physicians could be identified. Data collection was undertaken in line with European Pharmaceutical Marketing Research Association guidelines, so ethics committee approval was not required.

### 2.1 Participants

The physicians included in this study were those who made treatment decisions for ≥5 patients per week [if a primary care physician (PCP)], or ≥10 patients per week (if a specialist, including neurologists, geriatricians, and psychogeriatricians) with a diagnosis of MCI or dementia/AD. PCPs and specialists were invited to take part in the survey through specialized recruitment agencies. Patients were eligible to be included if they were 50 years or older and had a physician-confirmed diagnosis of MCI or a specified form of dementia, including AD. Exclusion criteria included having solely vascular dementia, dementia resulting from environmental factors such as traumatic brain injury or alcoholism, or current participation in a clinical trial at the time of data collection.

### 2.2 Study procedures

Detailed methodology has been described previously (21). Physicians completed an attitudinal physician survey and then patient record forms for approximately nine consecutively consulting patients with a diagnosis of MCI or dementia/AD. The physician survey assessed perceived reasons for diagnostic delays, key barriers to timely diagnosis, and the role of biomarker testing in MCI/AD dementia. Patient record forms captured demographics, clinical characteristics, symptoms at first consultation, consulting and diagnosing physicians, time to diagnosis, diagnostic tests, disease severity, and current treatment and management. For each patient record form completed by physicians, the corresponding patient was invited to provide a self-completion form reporting reasons for delaying their first consultation about their memory problems. Completion of these forms was voluntary, so data were not obtained from all patients.

This analysis focused only on patients whom physicians classified as having either “MCI” or “AD dementia.” For those with dementia due to AD, physicians assessed current disease severity as mild, moderate, or severe based on medical records, consultation findings, patient/family feedback, and clinical judgment. The Mini-Mental State Examination (MMSE) scores were used to assess severity at the first consultation when available.

For data harmonization, patient self-reported data (e.g., reasons for delaying their first consultation with a healthcare practitioner) were included only when a corresponding patient record form completed by the physician was available for analysis, ensuring that patient-reported information was analyzed only for patients for whom we also have physician-provided data. Additionally, all physicians completed the attitudinal survey before providing information on patients seen in clinical practice, allowing the comparison of physicians' self-reported attitudes toward management and testing with their actual clinical practice at an individual patient level.

### 2.3 Statistical methods

Descriptive statistics included means and standard deviations for continuous variables and frequencies with percentages for categorical variables. Medians and interquartile ranges were also reported. Missing data were not imputed, leading to variable base sizes. Analyses were conducted using STATA^®^ Version 17 (StataCorp LLC, College Station, Texas, USA).

## 3 Results

### 3.1 Physician and patient characteristics

A total of 94 physicians in Spain participated in the DSP, reporting data for 723 patients, and 102 patients' self-reported data. Of the physicians, 44.7% were PCPs, 38.3% neurologists, 7.4% geriatricians, and 9.6% other specialties; 77.2% of patients were seen in public and 22.8% in private settings (Supplementary Table S1). Physicians reported data for 723 patients with MCI or AD, whose sociodemographic and clinical characteristics are shown in Table 1. Patients had a mean (SD) age of 77.5 (7.7) years, and 54.1% were female. The diagnosis at the time of the survey was MCI for 37.3% of patients, and AD for 62.7%. In patients with an available MMSE score at the time of initial diagnosis (*N* = 501), MCI (MMSE score 27–28) was present in 10.4%, mild dementia (MMSE score 20–26) was found in 67.5%, and moderate or severe dementia (MMSE ≤ 19) in 22.2%. Most patients had a comorbidity (85.9%), with arterial hypertension (44.3%), anxiety (31.0%) and depression (28.1%), dyslipidemia (21.4%), and diabetes without chronic complications (14.2%) frequently reported. A total of 209 patients (33.7%) were taking anti-depressants as a comorbid-related treatment.

**Table 1:** Physician-reported demographics and clinical characteristics of patients with a current MCI or AD dementia diagnosis.

**Variable**	***N* = 723**
Age, mean (SD)	77.5 (7.7)
Sex (women), *n* (%)	391 (54.1)
Weight (kg), mean (SD)	71.6 (11.3)
**Current diagnosis**, ***N*** **(%)**, ***N** = **711***	
MCI - suspected AD	244 (34.3)
MCI - unknown AD	21 (3.0)
AD with mild dementia	89 (12.5)
AD with moderate dementia	229 (32.2)
AD with severe dementia	128 (18.0)
**MMSE score at initial diagnosis**, ***N*** **(%)**, ***N** = **501***	
Mild cognitive disease (MMSE = 27–28)	52 (10.4)
Mild dementia (MMSE = 20–26)	338 (67.5)
Moderate dementia (MMSE = 10–19)	100 (20.0)
Severe dementia (MMSE = 0–9)	11 (2.2)
**Comorbidities**^a^, ***N*** **(%)**, ***N*** = **621 (85.9%)**	
Arterial hypertension	320 (44.3)
Anxiety	224 (31.0)
Depression	203 (28.1)
Dyslipidemia	155 (21.4)
Diabetes without chronic complications	103 (14.2)
Hyperlipidemia	91 (12.6)
Back pain	82 (11.3)
Insomnia/sleep disorders	79 (10.9)
Osteoporosis	77 (10.7)
**Treatments for comorbidities**, ***N*** **(%)**, ***N** = **621***	
Anti-depressants^b^	209 (33.7)
Anti-platelet	54 (8.7)
Anti-coagulants	49 (7.9)

^a^Comorbidities diagnosed and present in ≥10% of patients. Multiple responses were possible.

^b^Includes SSRI/SNRI antidepressants, MAOI inhibitors, other anti-depressants.

AD, Alzheimer's disease; MCI, mild cognitive impairment; MMSE, mini mental state examination; SD, standard deviation.

### 3.2 Pre-diagnosis

**Figure 1:**
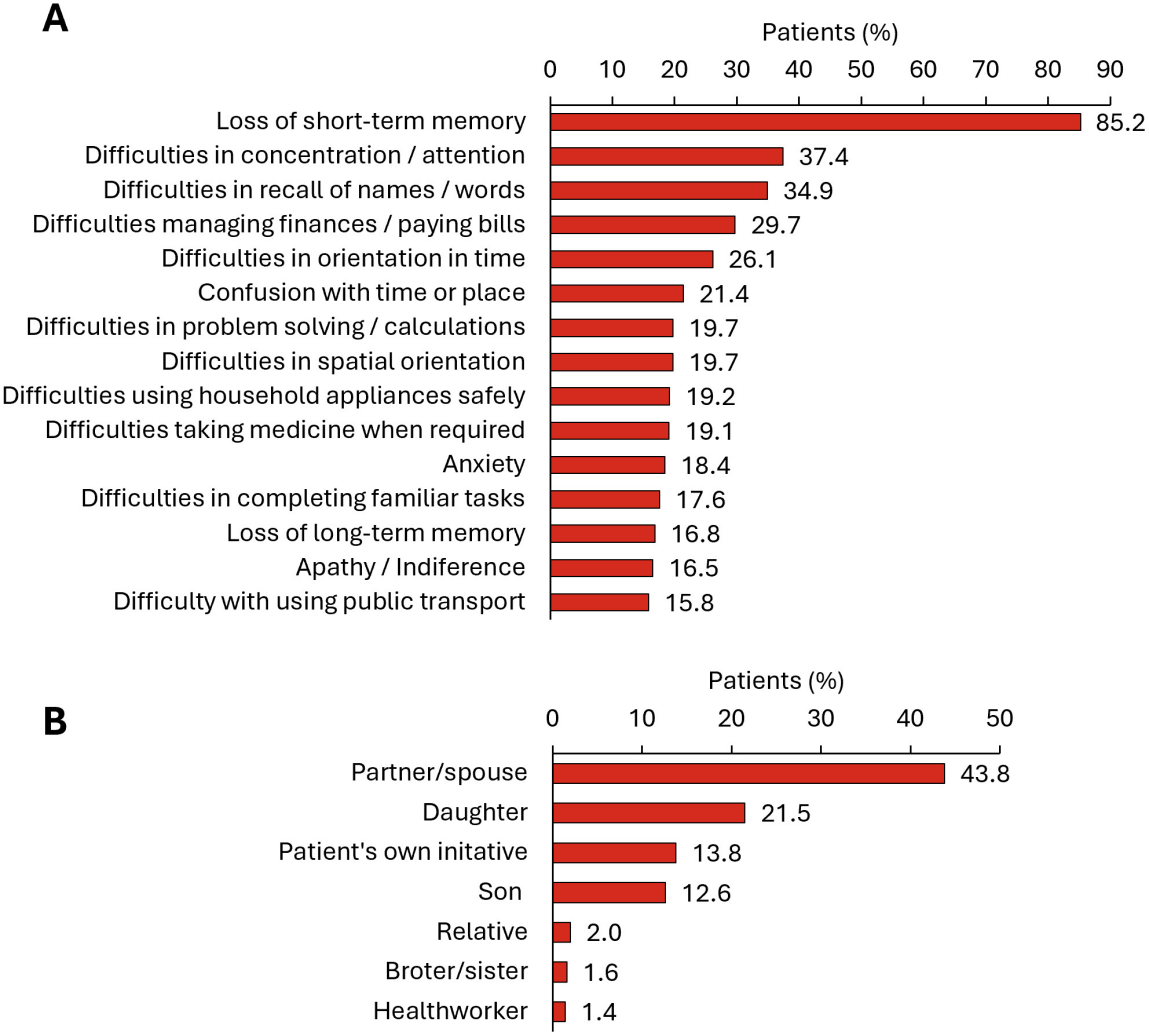
First MCI or dementia related consultation. **(A)** Symptoms prompting first consultation (list includes those symptoms reported by ≥15% of people with MCI or dementia) (*N* = 697); **(B)** Individual who first noticed the person's cognitive decline and prompted initial consultation (*N* = 708).

The most frequent symptoms that prompted patients to first consult with a physician (*N* = 697) were the loss of short-term memory (85.2% of patients), difficulties in concentration (37.4%), difficulties in recalling names and words (34.9%), difficulties managing finances and paying bills (29.7%), and other activities of daily life (Figure 1A). The individual who first noticed the person's cognitive decline and prompted initial consultation was generally the patient's partner or spouse (43.8%), followed by a daughter (21.5%) (Figure 1B). Only 13.8% of patients consulted for the first time on their own initiative.

**Figure 2:**
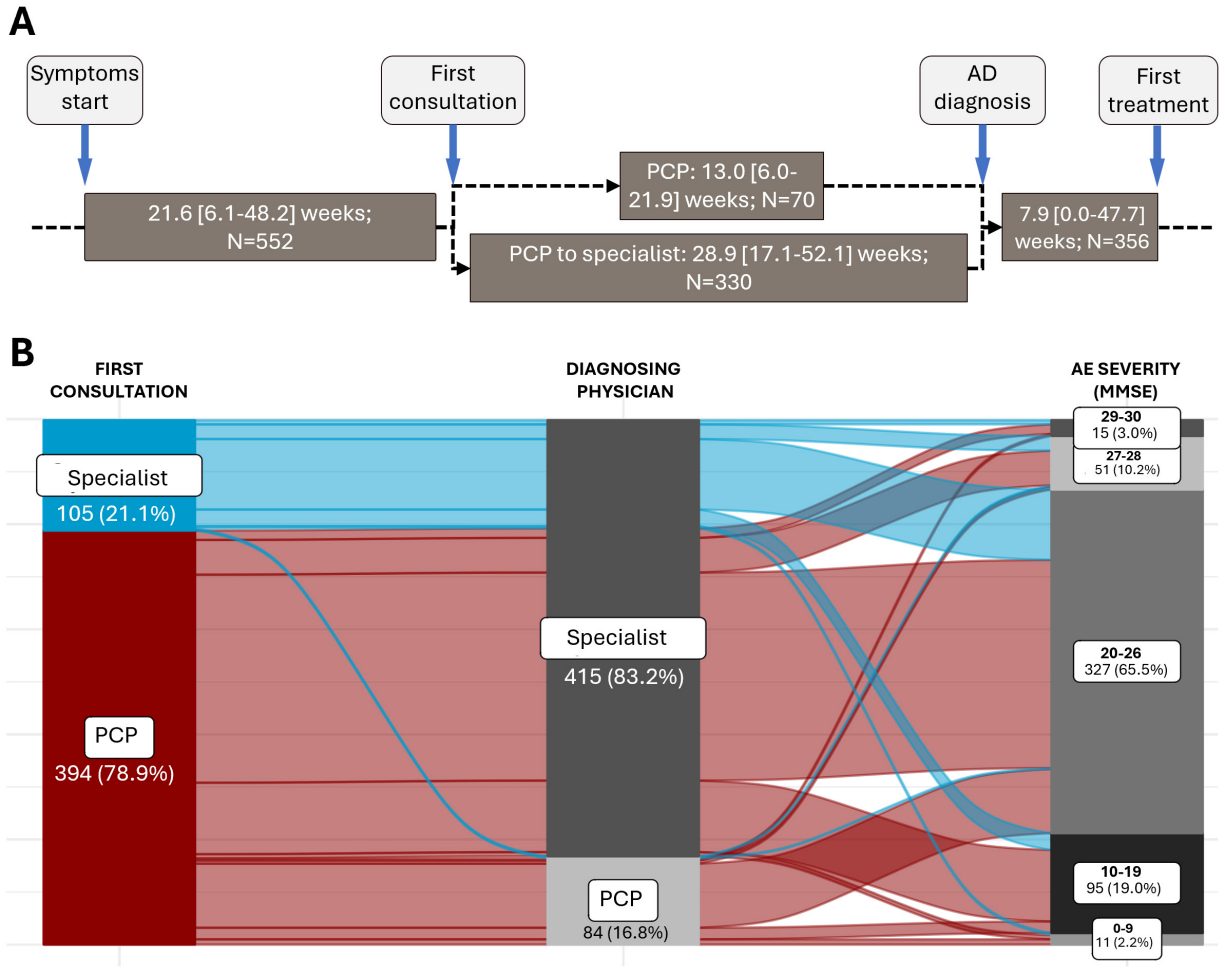
Timeline of AD diagnosis. **(A)** Timeline of the diagnostic process of people with MCI/AD. The “PCP to specialist” pathway indicates people who first consulted a PCP and are then referred to a hospital specialist for diagnosis. Values are median [IQR]. **(B)** Sankey graph representing the diagnostic journey of patients at different stages of AD severity according to the MMSE questionnaire. Only patients for whom all the information available in their history regarding the collected variables are shown (*N* = 499 patients).

**Figure 3:**
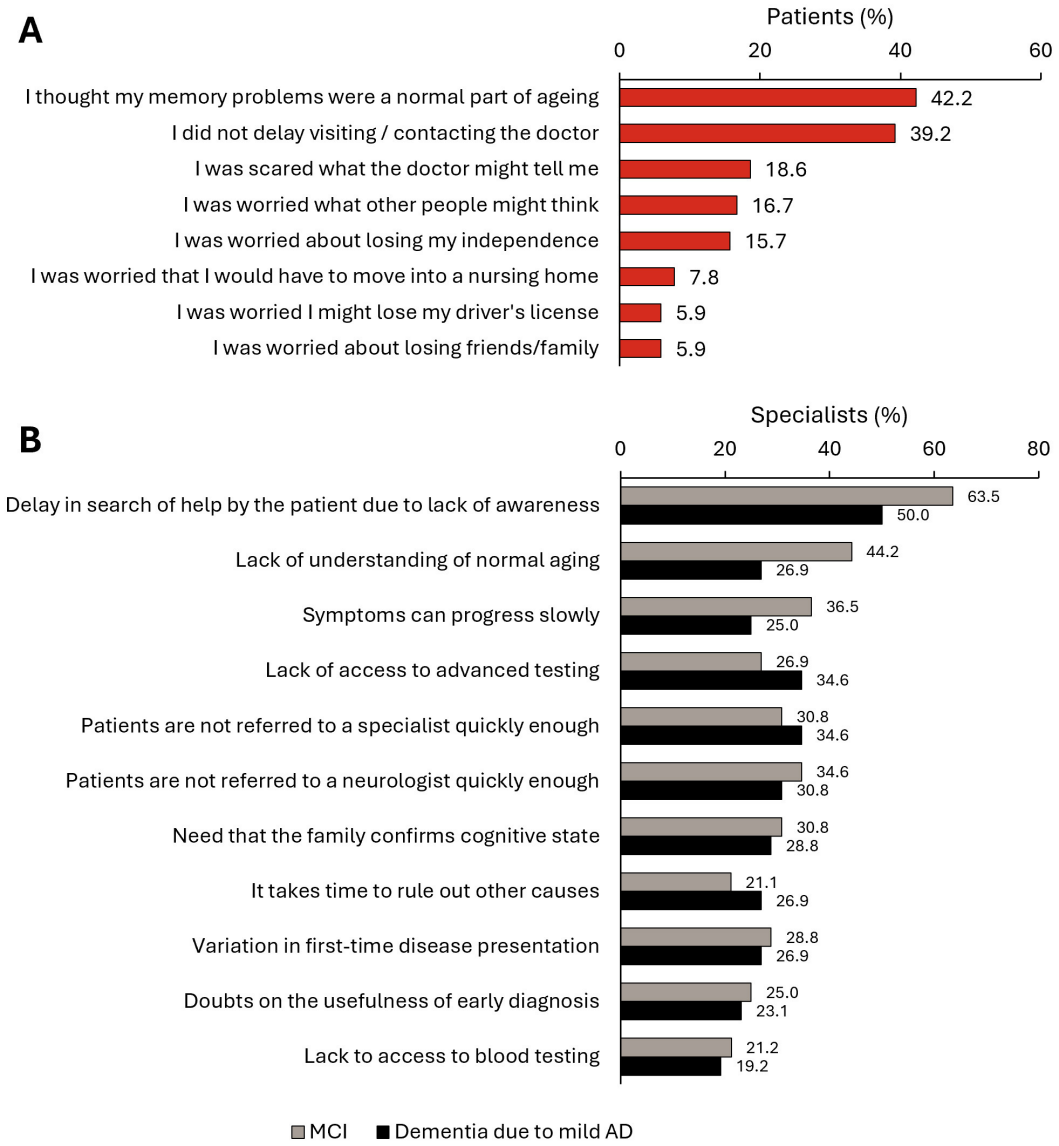
Delay in early diagnosis of dementia. **(A)** Reasons given by patients (*N* = 102) for delaying the first MCI-related consultation. **(B)** Main diagnostic barriers for early identification of patients with MCI and dementia due to mild AD according to specialists (*N* = 52).

The median (IQR) time since the symptoms started to the first consultation was 21.6 (6.1–48.2) weeks (Figure 2A). In this study, 78.9% of patients consulted first with a PCP, and 21.1% to a hospital specialist (Figure 2B). The main reasons given by patients (*N* = 102) for delaying the first consultation were thinking that their memory problems were a normal part of aging (42.2%), being afraid of the diagnosis (18.6%), being worried about what other people would think (16.7%), or being worried about losing independence (15.7%) (Figure 3A). According to specialists, the main diagnostic barriers for early identification of patients with MCI and dementia due to mild AD was also the patients' delay due to lack of awareness of symptoms or stigma (MCI: 63.5%; AD with mild dementia: 50.0%) and lack of understanding of normal aging process (44.2%; 26.9%) (Figure 3B).

### 3.3 Diagnosis

Although PCPs primarily conducted the first consultation, most patients were referred to neurologists or other hospital specialists. The diagnosing physician was a specialist for 83.2% of patients, and only 16.8% were diagnosed by a PCP (Figure 2B). Of patients diagnosed by a PCP, 86.9% had an MMSE score of 10–26 at diagnosis; for patients diagnosed by specialists, 84.1% had an MMSE score of 10–26, and 14.5% with MMSE 29–30 at diagnosis (data not shown). In patients who first consulted a PCP and were not diagnosed in the first consultation, the median time from the initial consultation to diagnosis differed if it was the PCP who diagnosed the patient [13.0 (6.0–21.9] weeks), or if the PCP referred the patient to a specialist, in which case the time to diagnosis extended to 28.9 [17.1–52.1] weeks (Figure 2A).

**Figure 4:**
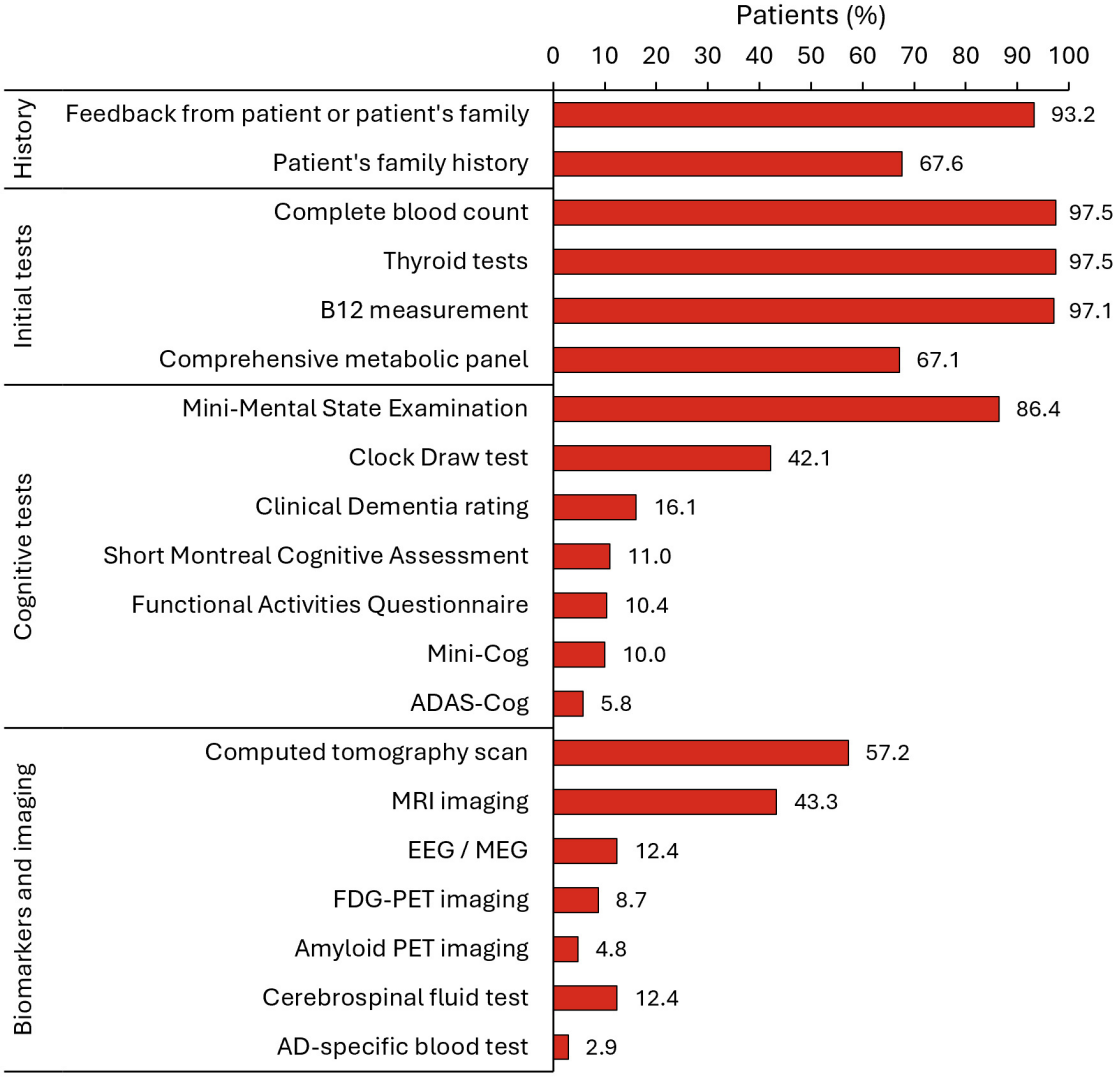
Specified tools that were used to aid in the diagnosis of AD (*N* = 691). AD, Alzheimer's disease; ADAS-Cog, Alzheimer's Disease Assessment Scale-Cognitive Subscale; EEG, electroencephalogram; FDG-PET, fluorodeoxyglucose-positron emission tomography; MEG, magneto-encegalogram; MRI, magnetic resonance imaging; PET, positron emission tomography.

During the diagnosis process, physicians assessed the medical history of the patient and feedback from the patient's family and/or friends, and performed cognitive tests, a complete blood count, thyroid tests, vitamin B12 measurements, and a comprehensive metabolic panel (Figure 4). Among the cognitive tests, the MMSE and the clock draw test were performed on 86.4% and 42.1% of patients, respectively. At diagnosis, most patients (>65%) had MCI or mild dementia due to AD (MMSE score 20–26) (Figure 2B).

Further testing during the diagnosis process included imaging and the use of biomarkers. Computed tomography (CT) scans were performed on 57.2% of patients, magnetic resonance imaging (MRI) in 43.3%, EEG/MEG in 12.4%, FDG-PET in 8.7%, amyloid PET imaging in 4.8%, CSF determination of AD biomarkers in 12.4%, and AD-specific blood biomarkers in 2.9% (Figure 4). Specialists reported that the mean (SD) time elapsed between their referral to patients undergoing the test were 73.1 (48.5) days for amyloid PET, 61.0 (40.0) days for volumetric MRI, 32.1 (29.9) days for a blood test of AD biomarkers, and 30.8 (28.3) days for a CSF test (Supplementary Table S2).

**Figure 5:**
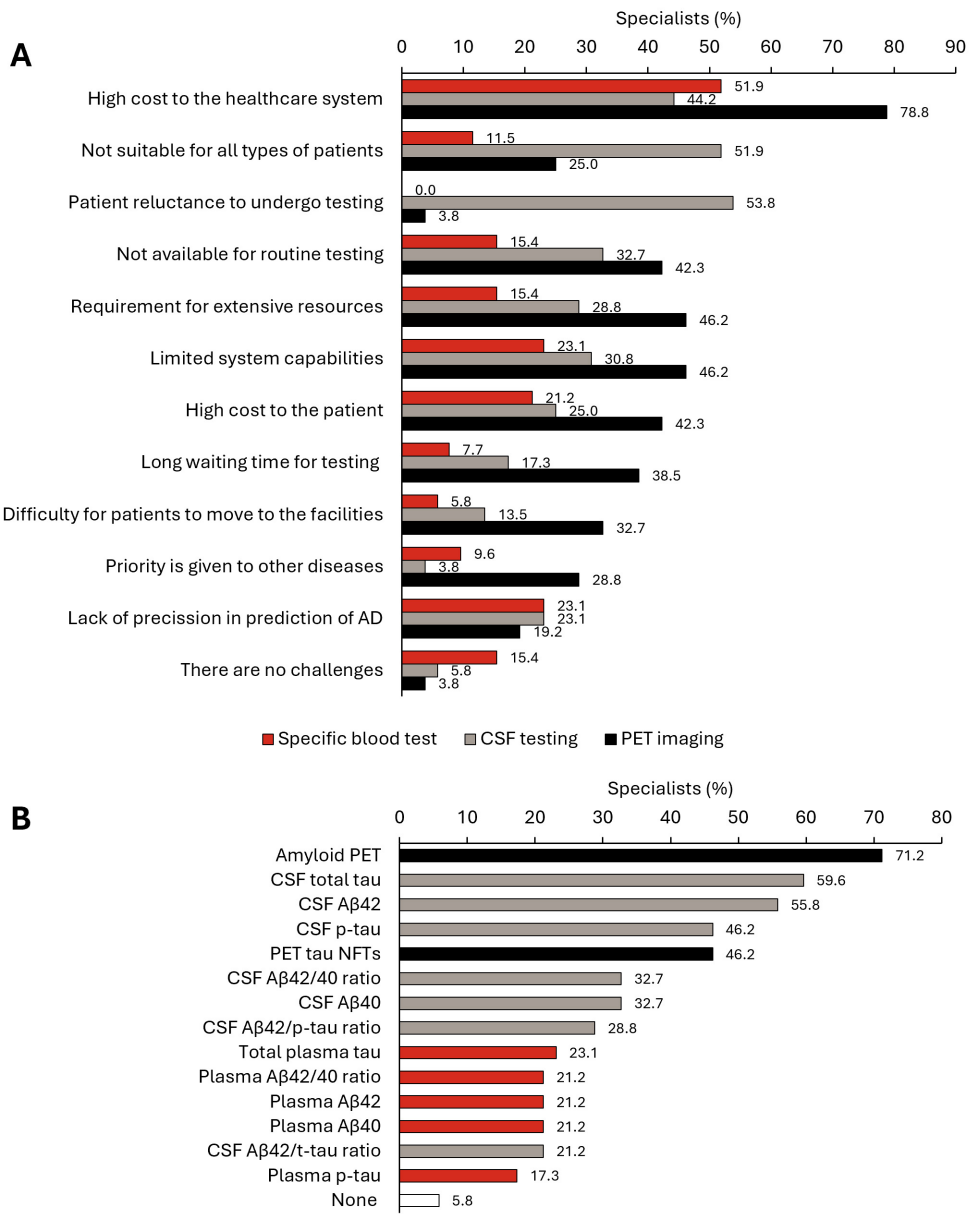
Use of biomarkers in routine clinical practice. **(A)** Views of specialists (*N* = 52) on various challenges in adopting precision biomarker testing in routine clinical practice. **(B)** Percentage of specialists (*n* = 52) who consider each specific biomarker during the AD diagnosis. AD, Alzheimer's disease; CSF, cerebrospinal fluid; NTFs, neurofibrilar tangles; PET, positron emission tomography.

The challenges faced by specialists when adopting AD biomarker testing during diagnosis in routine clinical practice are shown in Figure 5A. For AD specific blood tests, 51.9% of specialists believed that the biggest challenge was their costs to the healthcare system, and 23.1% thought that it has limited capabilities and precision in the prediction of AD. For CSF testing, the major challenges were the patient's reluctance to undergo the testing (53.8%), that it is not suitable for all types of patients (51.9%), and the high costs to the healthcare system (44.2%). Concerning PET imaging, the costs were also the main challenge (78.8%), followed by the requirement for resources and capabilities (46.2%) and the high cost to the patient (42.3%). Specialists considered that the most specific biomarkers for the identification of AD diagnosis were amyloid plaque (71.2%), CSF (total tau) (59.6%), and CSF (Aβ42) (55.8%) (Figure 5B). When asked about the future relevance of validated biomarkers in the identification of patients with AD with a clinical diagnosis of MCI, most specialists responded “important” (38.5%) or “extremely important” (48.1%) (Supplementary Figure S1A). On their plans to use various tests in the future, specialists indicated that they plan to use AD specific blood tests (85.4% of participants), CSF testing (34.1%), or PET imaging (40.4%) (Supplementary Figure S1B).

### 3.4 Treatment

**Figure 6:**
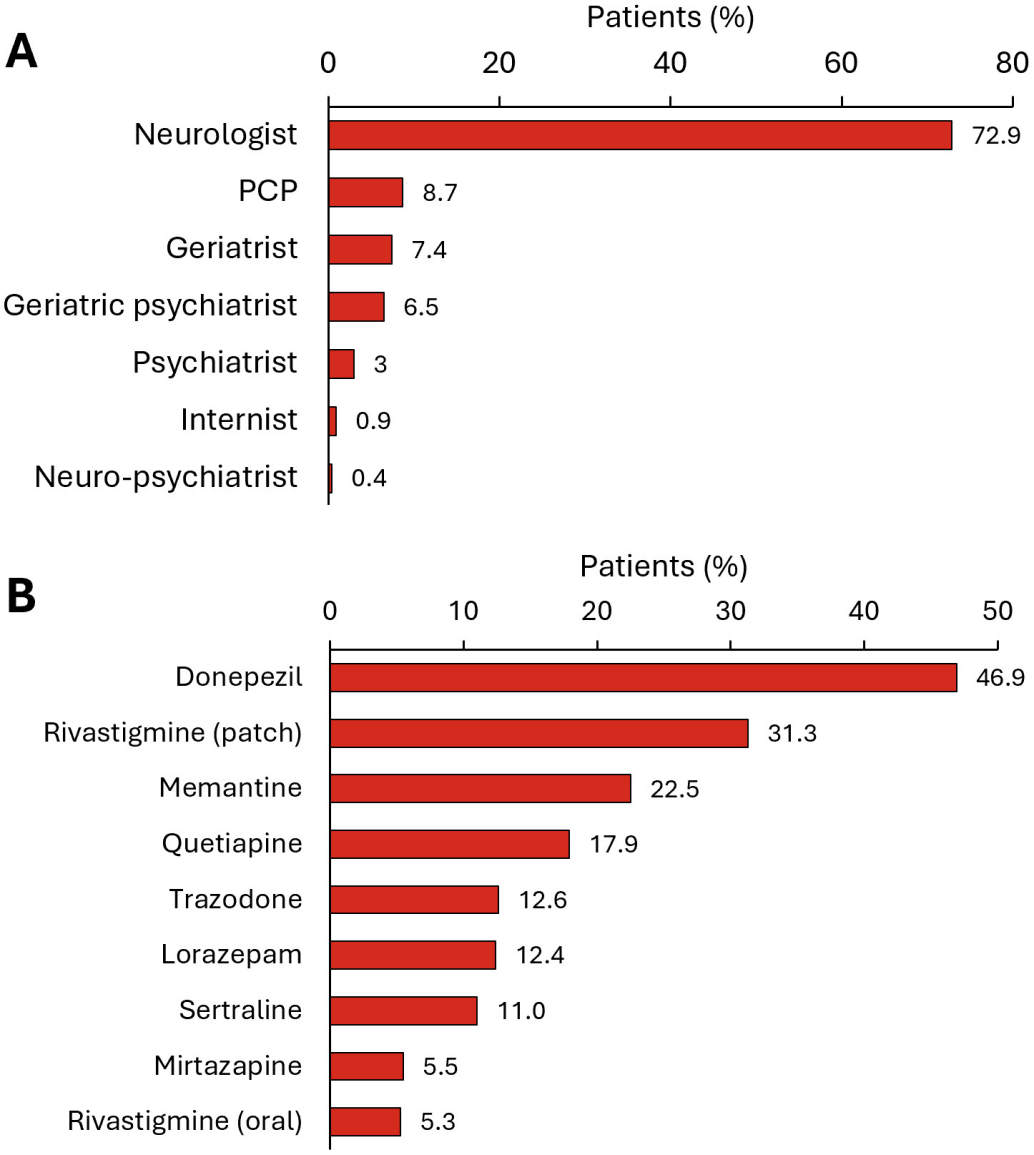
Treatment initiation in all patients and patients with MCI or AD with mild dementia. **(A)** Physician who initiated the patient's first treatment after a diagnosis of AD (*N* = 565 patients). **(B)** Treatment received in the first line (*N* = 565 patients). Only treatments administered to >5% of patients are shown.

Physicians who initiated treatment were mostly neurologists (72.9%). PCPs initiated treatment in only 8.7% of patients, followed by geriatricians (7.4%) and geriatric psychiatrists (6.5%) (Figure 6A). The most commonly administered treatments were donepezil (46.9% of patients), followed by rivastigmine (patches) (31.3%), and memantine (22.5%) (Figure 6B).

## 4 Discussion

The results of the large survey of Spanish physicians show that most people with MCI and dementia due to AD first see a PCP and are then referred and diagnosed by a hospital specialist (mainly a neurologist), who also initiated the treatment. Most patients were initially diagnosed in the mild dementia phase and a very small proportion of patients received a diagnosis utilizing biomarker tools during the assessment. However, most specialists consider the future use of precision biomarkers to be important for the diagnosis of early-stage AD and hope to incorporate blood-based biomarker testing into their clinical practice. Specialists perceived several challenges associated with the use of CSF-, PET-, or blood-based biomarkers in routine clinical practice, mostly related to the high costs to the healthcare system and the use of resources. According to specialists, the diagnostic process for people with MCI and AD could be accelerated by increased awareness of the disease, shorter referral times, and better access to specialized diagnostic services. Improving diagnosis is essential to optimize treatment as new amyloid-targeting therapies are expected to be used in the near future in clinical settings in Spain.

The characteristics of the patients reported by the participants in the study were consistent with previous studies of the Spanish population of people with MCI and AD, and highlight the high prevalence of comorbidities such as hypertension, depression and anxiety, diabetes, and hyperlipidemia (26–30). About one-third of the people attending the first consultation were already taking anti-depressants (30–32). In this regard, a recent study in Spain showed that people who seek help for cognitive problems reported higher levels of anxiety, depression, and concern about their perceived cognitive decline, compared to those who did not seek help (16).

In Spain, as in other countries, complaints related to memory or cognitive impairment are a common reason for consultation in primary care (33). The survey showed that short-term memory loss is the predominant symptom that drives people with MCI or AD to seek medical attention, usually prompted by their partner/spouse or daughter. Most patients initially consulted a PCP, who referred them to a hospital specialist, usually a neurologist, for diagnosis. As the first point of contact for patients experiencing cognitive decline, PCPs are in a unique position to recognize the early warning signs of AD, as their ongoing relationship with patients allows them to track cognitive changes over time, which can be essential for early intervention (6, 14). Also, PCPs can perform initial cognitive screenings and assessments, ruling out other possible causes of memory loss, such as vitamin deficiencies, depression, or medication side effects. PCPs provide continuous care by managing comorbid conditions, coordinating treatment plans, and offering support to caregivers (34). By advising caregivers on available resources and coping strategies, PCPs can be essential in improving the patient's quality of life (35).

Consistent with prior research, survey participants often estimated a substantial delay between the appearance of the first symptoms of cognitive impairment and the etiologic diagnosis of AD, especially when the diagnosis was conducted by hospital specialists (15, 20). This delay is in part due to the patient and/or the caregivers, who often attribute memory problems to the natural aging process, delaying the search for medical help and a diagnosis (36). Patients and families are often unaware that early diagnosis of AD is vital because it enables them to plan for the future, access appropriate medical treatments, and adopt lifestyle changes that may slow disease progression. However, the determinants of the delay in diagnosis are varied and complex, as shown in research in other countries, and include healthcare system barriers such as long waiting times for neurology appointments or insufficient training of PCPs in dementia diagnosis (15, 37–40). A prior survey in Spain showed that most PCPs considered diagnosis at the early stage of value, but that they did not have sufficient time to manage the patient (14).

Clinical assessment, based on patient and family medical history, conventional blood tests, and the MMSE brief cognitive assessment test, was the most common practice for AD diagnosis, as shown in prior studies (14, 20). Imaging with CT and MRI was also widely used (20, 41), but the use of AD-specific blood tests was very low. Blood tests for Alzheimer's disease are not yet widely available for clinical use in Spain. According to the specialists' perceptions, the main challenges for future routine adoption of AD specific blood tests would be the high costs to the healthcare system and a perceived lack of diagnostic precision for AD. Blood-based biomarker tests offer a less invasive, more accessible alternative, detecting key indicators like amyloid-beta and phosphorylated tau proteins (42, 43). In a recent position paper, the Spanish Society for Neurology advised that more data, training, and appropriate infrastructure are required for the use of blood-based biomarkers in general neurology clinics and in primary care (44). To generate confidence in the use of plasma biomarkers among clinicians, it is essential to understand the factors influencing plasma biomarker measurement and interpretation before incorporating them into routine clinical practice (45). In this sense, the vast majority of specialists in the survey considered the use of precision biomarkers important for the diagnosis of early AD, and plan on incorporating blood biomarker testing into their clinical practice in the future.

This study revealed that CSF testing was used in only 12.4% of patients and amyloid PET was used in 4.8%. In the case of CSF testing, the main challenges for routine adoption were the patient's reluctance to undergo testing, the fact that it is not suitable for all patients, and the costs to the healthcare system. The invasiveness of CSF testing and the associated patient concerns have been observed in other surveys in Spain and elsewhere (18, 20). In contrast, the challenges for the routine adoption of amyloid PET were mostly the costs for the healthcare system and its limited capacity (18). Nevertheless, the underuse of biomarkers of AD such as PET or CSF, which are mostly accessible to hospital specialists, is noteworthy, as these biomarkers could allow for more timely interventions in patients with AD. The recent development and commercialization of amyloid-targeting therapies will accelerate the need for an accurate, fast, and cost-effective diagnosis of patients with early-stage AD (MCI and mild dementia) (46).

Although for most patients the PCP is mainly responsible for identifying the patients, performing the initial screening and referring them to the specialist for diagnostic confirmation, the results of this study show that generally the physician initiating a treatment for dementia was the neurologist. The most common treatments were the acetylcholinesterase inhibitors donepezil and rivastigmine, followed by the NMDA receptor antagonist memantine, consistent with clinical guidelines (47), and previous studies of prescription and of antidementia drugs in Spain and elsewhere (48, 49). Other medications (antidepressants, antipsychotics, and anxiolytics) were also used to manage common neuropsychiatric comorbidities associated to AD like anxiety, depression, and sleep disturbances.

Survey-based studies, like the one presented here, have inherent limitations that should be considered when interpreting the results. First, the sample does not necessarily represent the proportion of specialists diagnosing or treating patients with AD. For example, the participating physicians worked in centers that managed a sufficient number of patients to provide the required patient record forms, and the dataset may not be representative of under-resourced centers. Also, the cross-sectional design of the DSP prevented any conclusions about causal relationships. Recall bias, a common limitation of surveys, might also have affected the responses of physicians. However, physicians had the ability to refer to the patients' records while completing the survey, thus minimizing the possibility of recall bias. The DSP is based on a pseudo-random, convenience sample of physicians; while minimal inclusion criteria governed the selection of physicians, participation was influenced by their willingness to complete the survey. Participating patients may not reflect the general AD dementia population since the DSP only includes patients who are actively consulting with their physician.

This study examined the real-world patient journey and barriers to timely diagnosis of MCI and AD dementia in Spain. The data from the study revealed that the delays in initial consultations were mainly due to lack of patient awareness about the normal course of aging, which led them to believe that their cognitive impairment was part of this process. Conversely, diagnostic delays stemmed from the time required for specialist referrals and scheduling of tests. AD-specific and validated biomarkers are used in a very low percentage of patients and waiting times for them are long. Enhancing patient awareness and utilizing accurate biomarker testing are essential to improving diagnosis and enabling the future initiation of treatments that may slow disease progression.

## Data Availability

The data that support the findings of this survey are the intellectual property of Adelphi Real World. All requests for access should be addressed directly to Sarah Cotton at sarah.cotton@adelphigroup.com. Sarah Cotton is an employee of Adelphi Real World.
