# Supplementary material for: The diagnostic pathway of Alzheimer's disease in real-world clinical practice in Spain: results from the Adelphi Dementia Disease Specific Programme™
Source: Front Neurol. 2026 Jan 20;16:1702805. doi: 10.3389/fneur.2025.1702805 (PMC12864097; doi:10.3389/fneur.2025.1702805)
Supplement: Supplementary file 1 [file Supplementary_file_1.docx]

***Supplementary Materials***

**Table S1.** Physician characteristics.

| **Variable** | **N=94** |
| --- | --- |
| Specialty |  |
| PCP | 42 (44.7) |
| Neurologist | 36 (38.3) |
| Geriatrician | 7 (7.4) |
| Other | 9 (9.6) |
| Consultation setting |  |
| Public office | 40.1 |
| Public hospital | 37.1 |
| Private office | 13.1 |
| Private hospital | 11.6 |

Abbreviation: PCP, primary care physician

**Table S2. Waiting time for AD diagnostic tests**

| **Test** | **Mean (days)** | **SD** | **IQR** | **N** |
| --- | --- | --- | --- | --- |
| Amyloid PET | 73.1 | 48.5 | 30-91 | 36 |
| Volumetric MRI | 61.0 | 40.0 | 30-91 | 41 |
| Blood test | 32.1 | 29.9 | 10-56 | 23 |
| CSF test | 30.7 | 28.3 | 14-30 | 38 |

Abbreviation: AD, Alzheimer’s disease; CSF, cerebrospinal fluid; IQR, interquartile range; MRI, magnetic resonance imaging; PET, positron emission tomography; SD, standard deviation


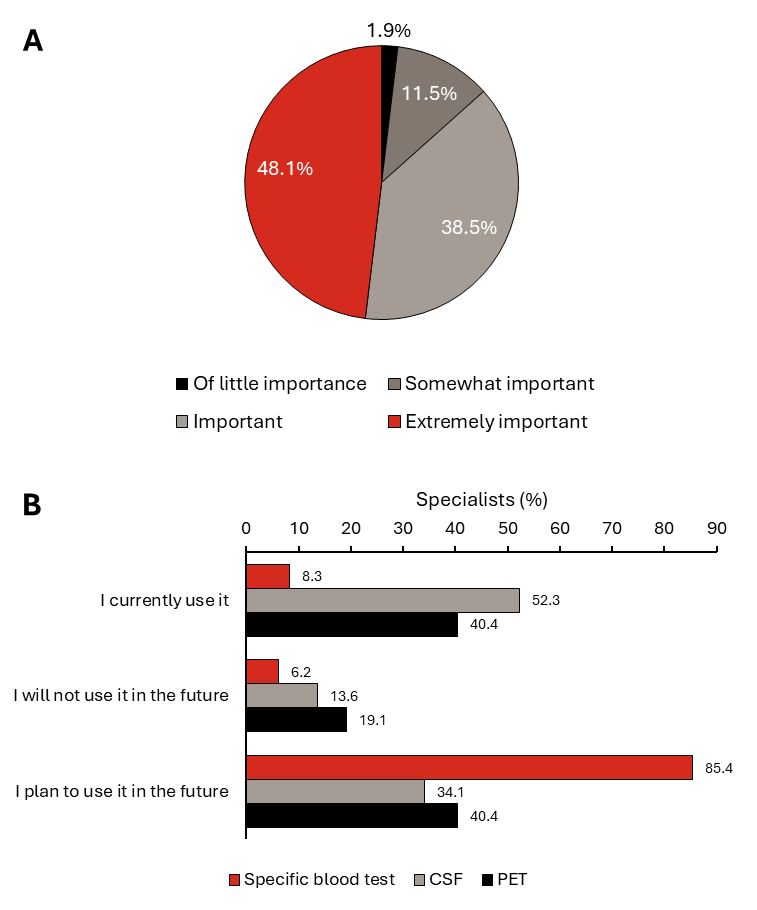


**Fig S1.** Future use of biomarkers among specialists (N=52). A. Future importance of using biomarkers to identify patients with AD and a clinical diagnosis of MCI.

Abbreviations: AD, Alzheimer’s disease; CSF, cerebrospinal fluid; MCI, mild cognitive disease; PET, positron emission tomography
